# Evaluation of enamel matrix derivative used alone or added to collagen membrane for tissue repair: in vivo animal study using a rat dorsal wound model

**DOI:** 10.1186/s40729-025-00635-5

**Published:** 2025-10-22

**Authors:** Julius Cezar Coelho Moraes, Filipe Rhuan Vieira de Sá Cruz, Lucas Novaes Teixeira, João Pedro Rangel-Coelho, Elizabeth Ferreira Martinez

**Affiliations:** 1 Division of Implantology, Faculdade São Leopoldo Mandic (SLMandic), Campinas, São Paulo, Brazil; 2 Division of Oral Pathology, Faculdade São Leopoldo Mandic (SLMandic), Campinas, São Paulo, Brazil; 3 Division of Cell Biology, Faculdade São Leopoldo Mandic (SLMandic), Campinas, São Paulo, Brazil; 4 Division of Cell Biology, Faculdade São Leopoldo Mandic, R. Dr. José Rocha Junqueira, 13,, 13045-610 Campinas, SP Brazil

**Keywords:** Gingival substitutes, Amelogenins, Collagen matrix, Periodontal regeneration

## Abstract

Gingival biotype is decisive for the outcome of rehabilitation treatments, and its impact on therapeutic prognosis is directly related to tissue fragility and marginal tissue recessions. This study aimed to evaluate the effect of enamel matrix derivative (EMD) and porcine collagen matrix, alone or combined, on wounds made on the rat dorsum. Wounds were created in the dorsum of 40 Wistar rats divided into 4 sample groups: G1 = clot, G2 = porcine collagen matrix (Straumann^®^ Mucoderm^®^), G3 = enamel matrix derivative (EMD, Emdogain^®^), G4 = porcine collagen matrix (Straumann^®^ Mucoderm^®^) and EMD (Emdogain^®^). The animals were euthanized on days 7 and 14, and their wound was evaluated histologically to assess wound closure and re-epithelialization, and to measure tissue inflammation. At 7 days, groups treated with EMD (G3 and G4) exhibited significantly lower inflammation scores compared to G1 and G2 (*p* < 0.05). At 14 days, all groups showed complete wound closure with no significant differences in inflammatory scores. However, G3 demonstrated a markedly greater epithelial thickness, with epithelial projections into the underlying connective tissue and evidence of keratin formation. Collagen fiber organization was also more evident in G3 and G4. Therefore, porcine collagen matrix, whether alone or combined, promoted tissue regeneration, and the combination of these substitutes with EMD, resulted in less tissue inflammation and greater epithelial thickness. These advantages emphasize their use for soft tissue augmentation and improvement.

**Supplementary Information:**

The online version contains supplementary material available at 10.1186/s40729-025-00635-5.

## Introduction

Dental implant surgeries are a reliable, predictable and efficient treatment, with high survival rates after 10 years of prosthetic loading [1, 2]. In general, dental implant failure may be mechanical or biological. Peri-implantitis is the most common cause of implant-related biological complications. Moreover, gingival recession and insufficient soft tissue can cause physiological damage, in addition to root hypersensitivity, and risk of tooth or implant loss [3]. Successful procedures in soft tissues require a combination of local, microbiological and cellular factors that include initial condition of the gingiva, biological capacity of the tissue, type of surgical technique, blood supply and regenerative potential of the periodontal tissue [4].

Therefore, both the quantity and quality of keratinized gingival tissue are important factors that influence the emergence and development of peri-implant lesions. The width of the keratinized mucosa around dental implants has been a relevant topic over the years. When keratinized gingiva measures less than 2 mm, even though factors such as bleeding on probing, pocket depth and plaque index may be important parameters of tissue health, and may cause inferior results, they may not be conclusive in terms of peri-implantitis emergence and development [3].

The gingival biotype is decisive to the outcome of rehabilitation treatments, and its impact on the therapeutic prognosis is directly related to tissue fragility and marginal tissue recession. Of like importance to the outcome is selecting the most appropriate surgical techniques for each case. Furthermore, improved gingival tissue, especially regarding thickness and the keratinized band, is tantamount to greater protection against marginal inflammation and trauma, hence enhancing the prognosis of the procedure [5].

Autogenous tissue grafts, including connective tissue grafts and free gingival grafts, have shown successful results in improving the soft tissue around teeth and implants. The free gingival graft is the gold standard. Although these techniques can promote a stable and effective gain of keratinized tissue, some factors, such as the limited amount of this tissue and increased postoperative morbidity of the donor site, are relevant clinical challenges [6, 7].

There are several techniques and biomaterials that have been developed to improve the keratinized mucosa, and augment the soft tissue around the teeth and dental implants. Dermal matrices are a clinical option enabling host cells to migrate to and penetrate the tridimensional framework of the matrix. In addition, they facilitate the formation of new blood vessels, and favor efficient tissue formation to augment the keratinized gingiva bands that promote periodontal and peri-implant health [8].

The porcine acellular dermal matrix under study (Straumann^®^ Mucoderm^®^) was created as an alternative to prevent the harvesting of palate mucosa, and secure results similar to those obtained for connective tissue formation, in terms of aesthetics and function [9]. Mucoderm^®^ has a large number of interconnected pores and a structure of native collagen, which assist in the migration of endothelial cells, and result in the formation of blood vessels, hence promoting fast tissue revascularization and integration. The matrix also enables the migration, adhesion and expansion of fibroblasts, as well as collagen matrix synthesis, thus allowing simultaneous tissue repair and matrix degradation. Full substitution of Mucoderm^®^ by the new host tissue occurs after 6 to 9 months [10].

In addition to the matrices, enamel matrix derivatives (EMDs, amelogenins) have been found to promote soft tissue regeneration. Amelogenins participate in several biological functions that are related to osteoblast and fibroblast regulation, and have been shown to increase the activation and activity of these cells. These proteins are commonly associated with embryo development, and play a role in tooth enamel formation and periodontal tissue regeneration. When applied to the donor sites for repair, they form an insoluble extracellular matrix precipitate with high hydroxyapatite and collagen affinity that allows them to interact with the surrounding cells, thereby activating and accelerating periodontal regeneration [11]. To this end, amelogenins have been used alone or combined with biomodulation tissue substitutes in clinical practice for root coverage or improvement of periodontal conditions [11, 12].

Therefore, bearing in mind the potential of both biomaterials being tested for clinical use in procedures involving gingival gain, this study aimed to evaluate their potential for tissue repair and modulation of dermal matrix inflammation, with or without EMD, in skin wounds on the dorsum of rats. The null hypotheses evaluated were that: (1) no difference in macroscopical and microscopical evaluation using EMD alone or associated with porcine collagen matrix compared to the other groups. 2) EMD used alone or associated with porcine collagen matrix would promote the same tissue inflammation than the other groups.

## Materials and methods

Forty Wistar rats *(Rattus norvegicus albinus)* were used in this study. The number of animals per group (*n* = 10) was based on prior published protocols using similar methodologies and was considered sufficient to detect relevant histological differences while minimizing animal use in accordance with the 3Rs principle (13). The research was carried out in compliance with the ARRIVE guidelines (Animal Research: Reporting of In Vivo Experiment), and with prior approval from the Research Ethics Committee for Animal Experimentation of Faculdade São Leopoldo Mandic (ethical protocol approval no. 2019/047). The animals were kept under controlled conditions of temperature (22 ± 2 °C) and lighting, with a 12-hour light-dark cycle, and with food and water *ad libitum*.

### Sample Groups

Inclusion criteria consisted of healthy, three-month-old male Wistar rats weighing approximately 300 g, with no signs of systemic illness or skin alterations. No exclusion criteria were applied, as all animals fulfilled the inclusion requirements and remained clinically stable throughout the study.

They were randomly divided into 4 groups (*n* = 10/each): G1, wound filled with clot; G2, wound covered with porcine collagen matrix (Mucoderm^®^, Botiss Dental Company, Zossen, Germany), G3 = wound filled with EMD (Emdogain^®^, Straumann manufacturing Inc., MA, USA), G4 = wound filled with EMD (Emdogain^®^, Straumann manufacturing Inc., MA, USA) and covered with porcine collagen matrix (Mucoderm^®^, Botiss Dental Company, Zossen, Germany). Random numbers were generated using the standard = RAND() function in Microsoft Excel, and allocation was concealed until the time of treatment by an independent investigator (F.R.V.S.C.). The surgeon (J.C.C.M.) was informed of the allocation only immediately before applying the material. All the groups were analyzed on days 7 and 14.

After fasting for 12 h, the animals received general anesthesia via intraperitoneal injection of 75 mg/kg ketamine hydrochloride (Dopalen Vetbrands, São Paulo, Brazil) and 10 mg/kg xylazine hydrochloride (Rompun Bayer, São Paulo, Brazil), ensuring sedation, muscle relaxation, and adequate analgesia for the surgical procedure. Postoperative pain management was based on daily monitoring of clinical signs (e.g., grooming, mobility, vocalization), and no additional analgesics were necessary, in accordance with the approved ethical protocol.

### Surgical Procedure

The rats were placed in a prone position, and trichotomy was performed on each dorsum, covering a 24 mm^2^ area (6 mm long x 4 mm wide), and positioned caudal to an imaginary line adjacent to the forelimbs. Antisepsis was performed using Povidone-iodine (PVP-I) with 1% active iodine (Rioquímica, São José do Rio Preto, São Paulo, Brazil). Demarcation was achieved by rotating the cutting edge of a 6-mm diameter metal punch (Kolplast, Itupeva, São Paulo, Brazil) at the center of the area prepared by trichotomy. A circular skin segment adjacent to the punch demarcation was then resected, and the incision was deepened until exposing the muscle fascia. After hemostasis was completed, the defects were distributed randomly among the 4 groups. G3 was the only group where the skin wound was covered with a transparent and sterile polyurethane film (Tegaderm™ Film, 3 M Health Care, St. Paul, MN, USA) to preserve the EMD gel and prevent its dispersion from the wound surface. The film was not replaced and typically remained in place for up to 4 days, after which it detached naturally due to fur regrowth and skin turnover. In both G2 and G4, the collagen matrices were stabilized using simple interrupted 6 − 0 nylon sutures to ensure proper adaptation to the wound bed.

After surgery, the rats were placed in individual cages, under temperature control (22 ± 2 °C), and with unrestricted food and movement. The wounds were monitored daily to assess animal health and ensure the absence of infection or complications. No standardized clinical wound healing score was applied.

The animals were euthanized on days 7 and 14 after surgery by CO_2_ inhalation. After dissection of the dorsa, the parts around the demarcated dorsum of the rats were fixed in 10% buffered formalin (Dinâmica^®^ Química Contemporânea LTDA, Indaiatuba, São Paulo, Brazil) and evaluated histologically on days 7 and 14.

### Macroscopic analysis

The parameters for evaluation were bleeding, secretion and wound closure. Visual assessment was the chosen method. Although visual inspection was used for the macroscopic assessment of wound status, the primary outcomes were determined through comprehensive histological and histomorphometric analyses, allowing for reproducible and detailed comparison among groups.

### Histological evaluation

Standardized and blinded histological evaluation was performed using predefined criteria to ensure reproducibility in assessing wound healing parameters. The samples removed were prepared for standard light microscopy. The specimens were embedded in histological paraffin (Dinâmica^®^ Química Contemporânea LTDA, Indaiatuba, São Paulo, Brazil) and 4-µm sections were performed transversely from the center of the defects, resulting in two sections per specimen at 10-µm intervals.

The samples were stained with hematoxylin-eosin (Dinâmica^®^ Química Contemporânea LTDA, Indaiatuba, São Paulo, Brazil) and then mounted on glass slides containing biologic mounting resin (Permount, Fisher Scientific, USA). Images of the slides were taken by a computerized image analysis system (AxioVision rel 4.8, Carl Zeiss), connected to a light microscope Axioskop 2 Plus (Carl Zeiss, Oberkochen, Germany). All histological and histomorphometric analyses were performed by a single experienced examiner (E.F.M.) who was blinded to the treatment groups, ensuring consistency and reducing potential inter-examiner variability.

The histological evaluation focused on detecting polymorphonuclear and mononuclear cells, vascular proliferation in granulation tissue and collagen fibers. A classification score was used to quantify the extent of inflammation of the defect area. The score ranged from 0 to 3, where 0 = absent, 1 = discrete (up to 25%), 2 = moderate (25–50%), and 3 = intense (more than 50%) [13, 14].

Re-epithelialization and wound closure were evaluated in all the groups as well. The wound was considered closed when the epithelial coating remained intact over the entire defect area. The data were presented in both absolute numbers and percentages for the different evaluation time periods.

### Statistical analysis

Descriptive analysis of the data was performed considering absolute and percentage frequencies. The data were also evaluated inferentially by statistical tests. Fisher’s exact test was used to evaluate significant differences among the groups, on a nominal scale. Ordinal scale data were assessed by the Mann-Whitney test for comparisons of euthanasia time points, and the Kruskal-Wallis test for comparisons among the groups. In the event of a significant result from the Kruskal-Wallis test, Conover’s post-hoc test was applied for pairwise comparisons. This method includes adjustment for multiple comparisons and controls the type I error rate through ranked pairwise contrasts, providing a suitable balance between stringency and statistical power in studies with limited sample size. The analyses were performed using IBM SPSS 25, with a significance level of 5%.

## Results

### Macroscopic evaluation

**Fig. 1:**
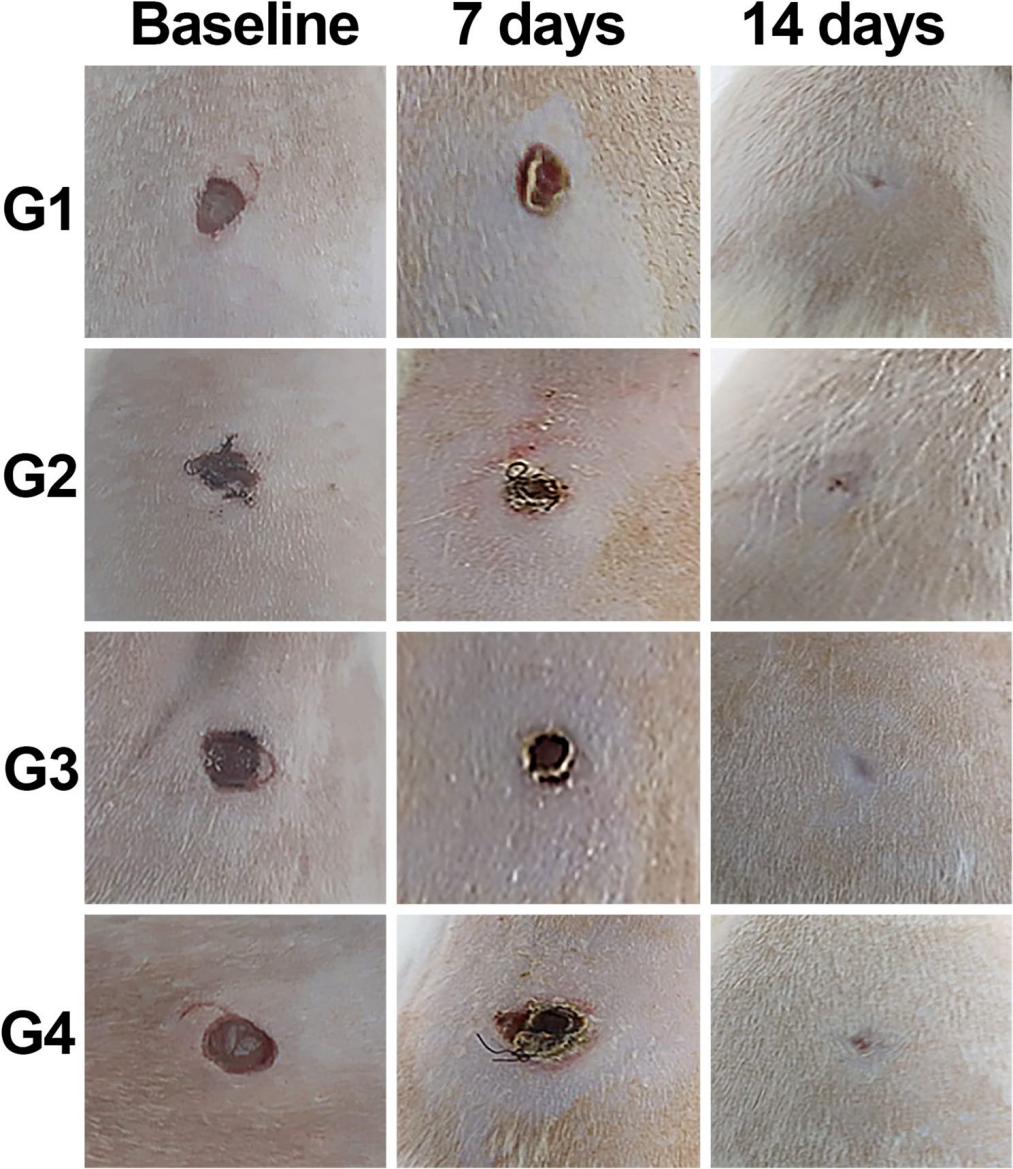
Representative images of the wounds at the initial time point (baseline), 7 and 14 days. Caption: **G1**: clot; **G2**: porcine collagen matrix (Mucoderm^®^); **G3** = enamel matrix derivative (Emdogain^®^), **G4** = enamel matrix derivative (Emdogain^®^) and covered with porcine collagen (Straumann^®^ Mucoderm^®^)

Macroscopic results of the wounds in each group are shown in Fig. 1. None of the wounds showed secretion, bleeding or granulation tissue on days 7 or 14. After 14 days, wound closure and re-epithelialization were observed in all the groups evaluated.

### Histological evaluation

**Fig. 2:**
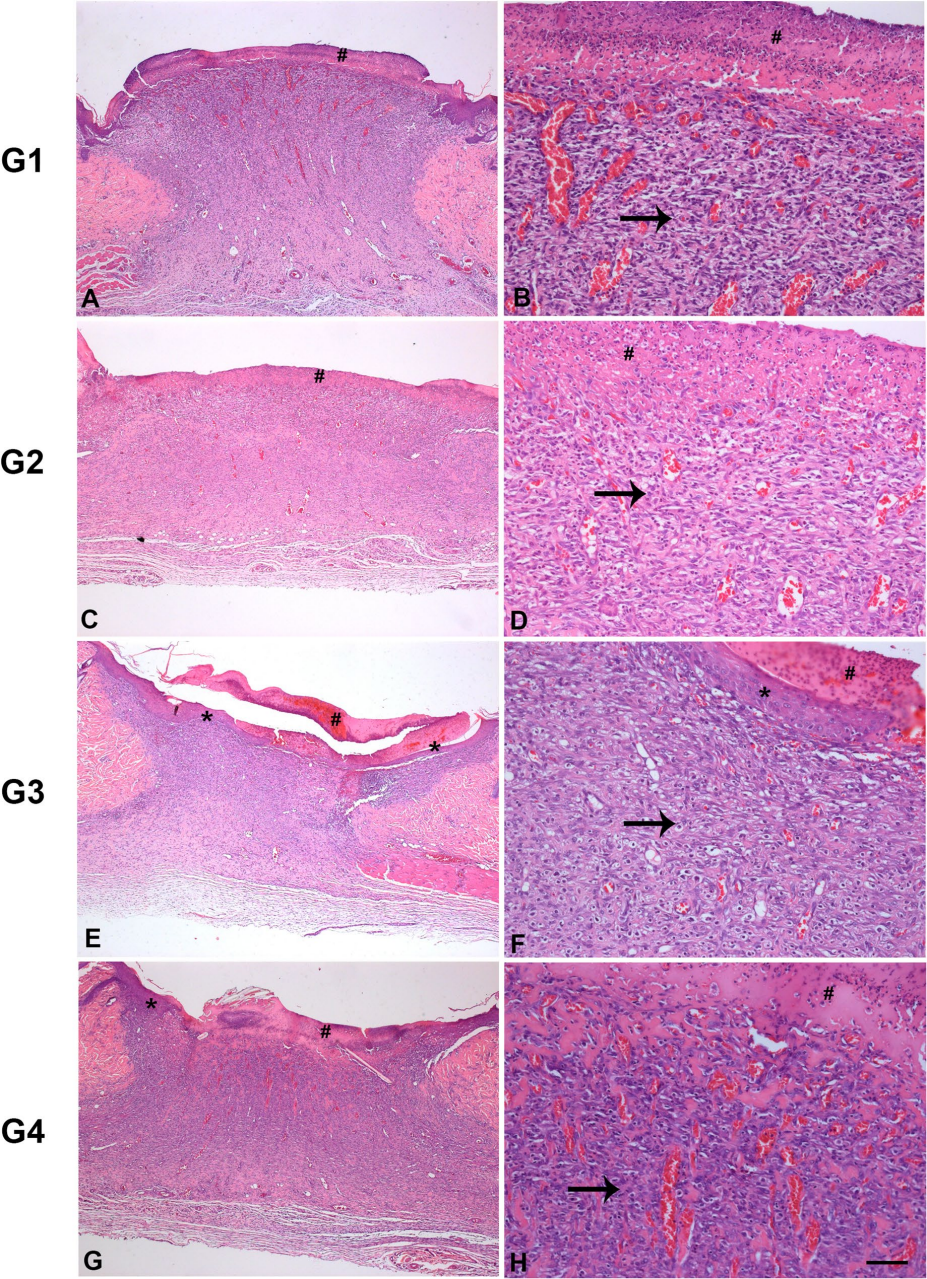
H&E-stained histological image representing the different groups at 7 days. Caption: *= epithelial tissue, arrow = granulation tissue, #= fibrin. Bar: **A**, **C**, **E**, **G** = 50 μm; **B**, **D**, **F**, **H** = 100 μm. G1: clot; G2: porcine collagen matrix (Mucoderm^®^); G3 = enamel matrix derivative (Emdogain^®^), G4 = enamel matrix derivative (Emdogain^®^) and covered with porcine collagen (Straumann^®^ Mucoderm^®^)

**Fig. 3:**
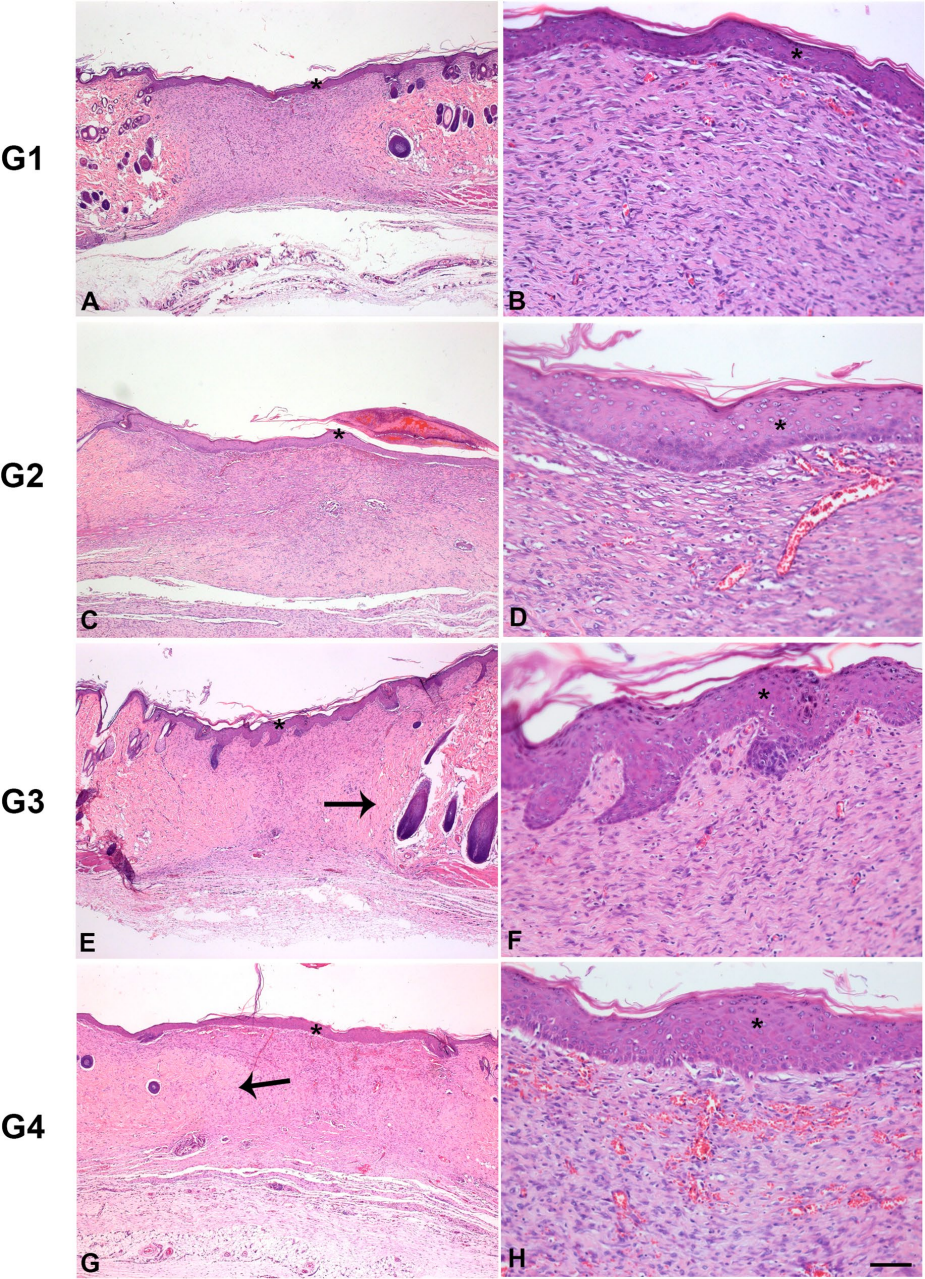
H&E-stained histological image representing the different groups at 14 days. Caption: *= epithelial tissue, arrow = collagen fibers. Bar: **A**, **C**, **E**, **G** = 50 μm; **B**, **D**, **F**, **H** = 100 μm. G1: clot; G2: porcine collagen matrix (Mucoderm^®^); G3 = enamel matrix derivative (Emdogain^®^), G4 = enamel matrix derivative (Emdogain^®^) and covered with porcine collagen (Straumann^®^ Mucoderm^®^)

The H&E-stained histological images representing the different groups at 7 and 14 days are shown in Figs. 2 and 3. At 7 days, no re-epithelialization of the wounds was observed in the groups evaluated, which were all covered with fibrin plugs. In addition, there was intense vascularization in granulation tissue and inflammatory infiltrate, predominantly mononuclear lymphocytic. However, a more advanced re-epithelialization was observed in G3, in relation to the other groups (Fig. 2).

After 14 days (Fig. 3), all groups showed wound closure with wound re-epithelialization and the start of granulation tissue maturation, which was characterized by a reduction in the number of blood vessels and the presence of a mild inflammatory infiltrate. In G3, the epithelial thickness was greater than in the other groups, with epithelial projections to the underlying connective tissue, and the presence of keratin. Additionally, collagen fibers were observed underlying the wound region in G3 and G4.

The microscopic results after treatment with biomaterials at different time points of analysis quantify the wound closure (Tables 1 and 2). At 7 days (Table 1), there was no wound closure in practically any sample, and no differences among the groups evaluated (*p* > 0.05). At 14 days (Table 2), almost all the samples were closed, with no differences among the groups evaluated (*p* > 0.05).

**Table 1:** Evaluation of the absolute number (percentage) of closed wounds at 7 days of analysis, treated with different materials

Group	No	Yes	Total	p value
	n (%)	n (%)	n (%)	
**G1**	5 (100)	0 (0)	5 (100)	p^1^ = 1.000
**G2**	5 (100)	0 (0)	5 (100)	
**G3**	4 (80)	1 (20)	5 (100)	
**G4**	5 (100)	0 (0)	5 (100)	

Caption: G1: clot; G2: porcine collagen matrix (Mucoderm^®^); G3 = enamel matrix derivative (Emdogain^®^), G4 = enamel matrix derivative (Emdogain^®^) and covered with porcine collagen (Straumann^®^ Mucoderm^®^)

^1^Fisher’s exact test

**Table 2:** – Evaluation of the absolute number (percentage) of closed wounds at 14 days of analysis, treated with different materials

Group	No	Yes	Total	p value
	n (%)	n (%)	n (%)	
**G1**	1 (20)	4 (80)	5 (100)	p^1^ = 1.00
**G2**	0 (0)	5 (100)	5 (100)	
**G3**	0 (0)	5 (100)	5 (100)	
**G4**	0 (0)	5 (100)	5 (100)	

Caption: G1: clot; G2: porcine collagen matrix (Mucoderm^®^); G3 = enamel matrix derivative (Emdogain^®^), G4 = enamel matrix derivative (Emdogain^®^) and covered with porcine collagen (Straumann^®^ Mucoderm^®^)

¹Fisher’s exact test

The analyses of inflammation quantification of wounds filled with different materials are presented in Table 3. At 7 days, a higher inflammation score was observed in G1 and G2, than in G3 and G4 (*p* < 0.05). At 14 days, no differences were observed among the groups evaluated (*p* > 0.05).

**Table 3:** – Median (minimum and maximum value) of inflammation scores in relation to the different groups and time points

Time	G1	G2	G3	G4	*p* value
**7 days**	3.0 (3.0; 3.0) Aa	3.0 (3.0; 3.0) Aa	2.0 (2.0; 3.0) Ba	2.0 (2.0; 3.0) Ba	p^1^ = 0.043
**14 days**	1.0 (1.0; 1.0) Ab	1.0 (1.0; 1.0) Ab	1.0 (1.0; 1.0) Ab	1.0 (1.0; 1.0) Ab	p^1^ = 1.000
**p value**	p^2^ = 0.008	p^2^ = 0.008	p^2^ = 0.008	p^2^ = 0.008	

Caption: G1: clot; G2: porcine collagen matrix (Mucoderm^®^); G3 = enamel matrix derivative (Emdogain^®^), G4 = enamel matrix derivative (Emdogain^®^) and covered with porcine collagen (Straumann^®^ Mucoderm^®^)

¹Kruskal-Wallis exact test with Conover comparisons

²Mann-Whitney test

Different capital letters indicate significant differences among the groups for each analytical time point (horizontal). Different lower-case letters indicate significant differences for each group for different analytical time points (vertical)

## Discussion

The self-repair capacity of gingival and osseous tissues depends on the magnitude and anatomy of the defect. However, the potential of these tissues is limited, and insufficient to meet long-term demands. In clinical practice, significant tissue deficiencies often occur after tooth element loss, and usually require regeneration procedures to improve the aesthetics and function of implant-supported rehabilitations [15]. In addition, connective tissue enhancement should be considered, particularly in cases of high risk of recession in thin gingival biotypes with buccal bone thickness less than 0.5 mm [16].

Soft tissue augmentation and/or conservation are often needed, but the additional morbidity caused by the harvesting of autogenous tissue grafts could dissuade against procedures involving soft tissue manipulation, especially extraction, implantation, and bone and soft tissue reconstruction in a single intervention [17]. Therefore, collagen matrices and healing modulators have become relevant for the conservation and improvement of protective periodontal, and especially peri-implant, tissue conditions [6, 18–21]. However, when compared to autogenous connective tissue grafts, collagen matrix revealed inferior clinical outcomes in root coverage procedures, due to the lack of cell-activating substances [22], raising doubts about its efficacy in periodontal regeneration procedures.

Given the different therapeutic options, this study evaluated the tissue repair potential in uniform wounds of two biomaterials, porcine collagen matrix (Straumann^®^ Mucoderm^®^) and EMD (Emdogain^®^), used alone or combined. The results revealed the occurrence of tissue regeneration in all the groups, evidenced by epithelial closure and collagen fibers in the adjacent connective tissue, especially after 14 days of evaluation. In addition, EMD alone or combined with the collagen matrix promoted greater epithelial thickness, supporting the partial rejection of the first null hypothesis.

Mucoderm^®^ is a resorbable matrix with a porous structure consisting of type I collagen, which stimulates tissue augmentation by creating a three-dimensional space for blood clot formation and tissue maturation, thus acting as a protective framework and barrier for the receiving area [23]. This biomaterial also promotes stable tissue integration, making it an important option for grafting [24–27]. Type I collagen is an important tissue constituent protein associated with periodontal health [28, 29]. In situations of periodontal defects, it promotes normal repair, characterized by connective tissue rich in blood vessels and collagen fibers, and by fibroblastic cells permeating the collagen matrix framework [30]. The results of this study indicated repair of the wounds covered with Mucoderm^®^, evidenced by epithelial closure and a large number of blood vessels and collagen fibers in the tissue repair regions. Note, however, that a higher inflammation score was observed for this group at 7 days, than for the EMD-treated groups at the same time point. These findings support the rejection of the second null hypothesis.

This highly inflammatory response may be related to the structure of the porcine collagen matrix tested, considering that the absence of a chemical treatment to increase the cross-links would lead to fast biodegradation and loss of structural integrity. Such a structural disorder in the collagen matrix occurs due to the activity of collagenase secreted by local cells, which triggers inflammatory cellular taxis to the grafted bed, and increases local inflammation to levels greater than those of earlier periods [31–33].

EMDs (Emdogain^®^) consist mainly of amelogenin, and promote chemotaxis and colonization of gingival fibroblasts [34–37], inducing periodontal regeneration [11, 38–40], as well accelerating wound closure [41]. Furthermore, in vitro studies have shown that EMD induces the expression of transforming growth factor-β (TGF-β), bone morphogenetic proteins (BMP), vascular endothelial growth factor (VEGF), and fibroblast growth factor 2 (FGF-2) [42], which are important for soft tissue healing. Although these growth factors were not directly investigated in the present study, the microscopic findings—such as the advanced re-epithelialization and increased collagen deposition observed in the EMD-treated groups—may be explained by a probable upregulation of these mediators, as reported in previous studies [12, 43].In addition, more collagen fibers were observed at the epithelial thickness, with projections to the underlying connective tissue in the groups treated with EMD (G3 and G4), especially at 14 days of evaluation. This outcome demonstrates the property of EMD to heal periodontal wounds, and indicates its clinical use in treating gingival recessions for greater formation of keratinized tissue [44, 45].

Another finding of this study was that the groups treated with EMD had more mature granulation tissue with less inflammation after 7 days (*p* < 0.05). Granulation tissue formation is critical during wound healing because it provides a suitable matrix for the wound closure by epithelialization [46]. Research has shown that EMD decreases the expression of pro-inflammatory cytokines (IL-4) and the secretion of VEGF by fibroblasts, hence playing an important role in decreasing inflammation [42, 43]. In addition to modifying the inflammatory process, there is evidence that when EMD is combined with Mucoderm, it increases endothelial cell proliferation, which may reflect an improvement in the formation of new blood vessels around and through the collagen tissue matrix, which may contribute to the beneficial wound healing effects with EMD [8]. Further studies are required to determine whether these biomaterials are capable of promoting tissue remodeling in a beneficial way in terms of clinical outcomes.

One limitation of the present study is the use of a dorsal skin wound model to evaluate biomaterials intended for oral soft tissue regeneration. Although this model offers advantages such as reproducibility, ease of surgical access, and reduced risk of contamination, it does not fully replicate the anatomical and histological characteristics of the oral mucosa. Nevertheless, this model has been used in previous studies to investigate the biological effects of enamel matrix derivative and collagen matrices on tissue repair [47, 48]. Therefore, the results presented here should be interpreted with caution when translated to clinical scenarios. Future studies using intraoral models, such as palatal or buccal mucosa wounds, are recommended to validate the translational relevance of these findings.

Therefore, the results of this study revealed that porcine collagen matrix, whether alone or combined, promoted tissue regeneration, and the combination of these substitutes with EMD, resulted in less tissue inflammation and greater epithelial thickness. These advantages emphasize their use for soft tissue augmentation and improvement. From a clinical perspective, these findings support the the potential use of enamel matrix derivative and porcine collagen matrices as effective and less invasive alternatives for soft tissue regeneration. Their ability to enhance epithelial healing and modulate inflammation suggests their applicability in procedures such as root coverage and peri-implant soft tissue enhancement, potentially reducing the need for autogenous grafts and associated donor site morbidity. However, further studies—particularly clinical trials and molecular investigations—are necessary to confirm these benefits and to elucidate the underlying biological mechanisms.

## Electronic supplementary material

Below is the link to the electronic supplementary material.

Supplementary Material 1

Supplementary Material 2

Supplementary Material 3

## Data Availability

No datasets were generated or analysed during the current study.
