# Supplementary material for: Evaluation of enamel matrix derivative used alone or added to collagen membrane for tissue repair: in vivo animal study using a rat dorsal wound model
Source: Int J Implant Dent. 2025 Oct 22;11:66. doi: 10.1186/s40729-025-00635-5 (PMC12545986; doi:10.1186/s40729-025-00635-5)
Supplement: Supplementary file 2 — Supplementary Material 2 [file 40729_2025_635_MOESM2_ESM.pdf]

EDITORIAL CERTIFICATION

TO WHOM IT MAY CONCERN

This document attests that the manuscript below was edited for proper English language usage, grammar, punctuation, spelling and style by the undernamed, a native English-speaking copyeditor, B.A. Hofstra University, NY, and owner of NCristina Martorana Traduções S/C Ltda. [CNPJ: 04.959.659/0001-20]. Neither the research content nor the author's/authors' intentions were altered in any way during the editing process.

Should there be any questions, please contact: ncris.mart@uol.com.br.

Manuscript title: Evaluation of enamel matrix derivative used alone or added to collagen membrane for tissue repair on the rat dorsum

Corresponding author: Elizabeth Ferreira Martinez

Co-authors: Julius Cezar Coelho Moraes, Filipe Rhuan Vieira de Sá Cruz, Lucas Novaes Teixeira, João Pedro Rangel-Coelho

Date: January 1, 2025

Copyeditor: Nancy Cristina Martorana
