# Supplementary material for: Evaluation of enamel matrix derivative used alone or added to collagen membrane for tissue repair: in vivo animal study using a rat dorsal wound model
Source: Int J Implant Dent. 2025 Oct 22;11:66. doi: 10.1186/s40729-025-00635-5 (PMC12545986; doi:10.1186/s40729-025-00635-5)
Supplement: Supplementary file 3 — Supplementary Material 3 [file 40729_2025_635_MOESM3_ESM.docx]

**Title page**

**Evaluation of enamel matrix derivative** **used alone or added to collagen membrane for tissue repair on the rat dorsum**

Julius Cezar Coelho Moraes, DDS, MSc, PhD^a^, Filipe Rhuan Vieira de Sá Cruz, DDS, MSc^a^, Lucas Novaes Teixeira, DDS, MSc, PhD^b^, João Pedro Rangel-Coelho, DDS^c^, Elizabeth Ferreira Martinez, DDS, MSc, PhD^d*^

^a^ Division of Implantology, Faculdade São Leopoldo Mandic (SLMandic), Campinas, São Paulo, Brazil

^b^ Professor, Division of Oral Pathology, Faculdade São Leopoldo Mandic (SLMandic), Campinas, São Paulo, Brazil

^c^ Assistant, Division of Cell Biology, Faculdade São Leopoldo Mandic (SLMandic), Campinas, São Paulo, Brazil

^d^ Professor, Division of Cell Biology, Faculdade São Leopoldo Mandic (SLMandic), Campinas, São Paulo, Brazil

***Corresponding Author:**

Elizabeth Ferreira Martinez, Professor, Division of Cell Biology, Faculdade São Leopoldo Mandic

R. Dr. José Rocha Junqueira, 13, Campinas, SP, Brazil - 13045-610

Phone: +55 19 3211-3600

Email: [elizabeth.martinez@slmandic.edu.br](mailto:elizabeth.martinez@slmandic.edu.br) or [dr.efmartinez@gmail.com](mailto:dr.efmartinez@gmail.com)

**Authors’ Contributions**

**Conceptualization**: Julius Cezar Moraes, Elizabeth Martinez; **Methodology**: Julius Cezar Moraes, Filipe Rhuan Cruz, João Pedro Rangel-Coelho; **Formal analysis**: Julius Cezar Moraes, Filipe Rhuan Cruz, Elizabeth Martinez; **Investigation**: Julius Cezar Moraes, Filipe Rhuan Cruz, João Pedro Rangel-Coelho, Elizabeth Martinez; **Supervision**, Lucas Teixeira, Elizabeth Martinez; **Visualization**: Julius Cezar Moraes, Filipe Rhuan Cruz, Lucas Teixeira, Elizabeth Martinez; **Writing—original draft**: Julius Cezar Moraes, Filipe Rhuan Cruz, Elizabeth Martinez; **Writing—review & editing:** all authors.
